# Tailoring gut immune responses with lipoteichoic acid-deficient *Lactobacillus acidophilus*

**DOI:** 10.3389/fimmu.2013.00025

**Published:** 2013-02-06

**Authors:** Yaíma L. Lightfoot, Mansour Mohamadzadeh

**Affiliations:** ^1^Department of Infectious Diseases and Pathology, College of Veterinary Medicine, University of FloridaGainesville, FL, USA; ^2^Division of Gastroenterology Hepatology & Nutrition, Department of Medicine, College of Medicine, University of FloridaGainesville, FL, USA

**Keywords:** *Lactobacillus acidophilus*, lipoteichoic acid, S-layer proteins, gut inflammation, dendritic cells, immune regulation

## Abstract

As highlighted by the development of intestinal autoinflammatory disorders when tolerance is lost, homeostatic interactions between gut microbiota, resident immune cells, and the gut epithelium are key in the maintenance of gastrointestinal health. Gut immune responses, whether stimulatory or regulatory, are dictated by the activated dendritic cells (DCs) that first interact with microorganisms and their gene products to then elicit T and B cell responses. Previously, we have demonstrated that treatment with genetically modified *Lactobacillus acidophilus* is sufficient to tilt the immune balance from proinflammatory to regulatory in experimental models of colitis and colon cancer. Given the significant role of DCs in efficiently orchestrating intestinal immune responses, characterization of the signals induced within these cells by the surface layer molecules, such as lipoteichoic acid (LTA), and proteins of *L. acidophilus* is critical for future treatment and prevention of gastrointestinal diseases. Here, we discuss the potential regulatory pathways involved in the downregulation of pathogenic inflammation in the gut, and explore questions regarding the immune responses to LTA-deficient *L. acidophilus* that require future studies.

## INTRODUCTION

The gastrointestinal tract possesses a highly specialized immunologic system comprised of both innate and adaptive immune components. These defense systems act in concert to maintain a state of alertness or physiological inflammation in the gut that enables the recognition and clearance of invading pathogens while remaining tolerant to the commensal microbiome (Sansonetti, 2004). By virtue of their antigen processing and presenting abilities, dendritic cells (DCs) are at the forefront of intestinal immune responses (Chang et al., 2012). DCs in the lamina propria constantly sample an array of food and microbial antigens and present them to resident T cells. Under steady state conditions, intestinal DCs induce the development of Th1 and Th17 effector T cells; however, at the same time, a specialized subset of regulatory CD103^+^ DCs promote the generation of induced regulatory T cells (iTregs; Siddiqui and Powrie, 2008) that prevent exacerbated Th1 and Th17 effector responses, and thus limit collateral tissue damage. Tregs express the transcription factor FoxP3 and suppress proinflammatory immune responses through the production of anti-inflammatory cytokines, including interleukin (IL)-10 and transforming growth factor-beta (TGF-β), and the surface expression of inhibitory molecules, such as cytotoxic T lymphocyte antigen 4 (CTLA-4) and lymphocyte activation gene-3 (LAG-3; Huang et al., 2004; Li et al., 2007; Rubtsov et al., 2008; Wing et al., 2008; Bos and Rudensky, 2012). Indeed, the transfer of total CD4^+^CD25^+^ Tregs efficiently mitigated established colitis in an experimental model of the disease (Mottet et al., 2003), and a deficiency of this cell population has been found in patients with ulcerative colitis (Takahashi et al., 2006). Although these studies highlight the role of thymic-derived or natural Tregs, subsequent studies have emphasized the importance of iTregs for disease resolution (Haribhai et al., 2009). Therefore, the induction of peripheral Tregs by regulatory DCs in the gut seems to be particularly crucial for microbial coexistence and colonic health. In support of this notion, colonic Tregs were found to express T cell receptor (TCR) repertoires that were distinct from those found on Tregs from other organs and were also specific for antigens encoded by commensal bacteria (Lathrop et al., 2011).

In addition to the aforementioned regulatory immune cells, and equally important for gut immune homeostasis is the composition of the gut microbiota (Round and Mazmanian, 2009; Consortium, 2012; Holmes et al., 2012). Recent elegant studies have contributed to our understanding of intestinal immune modulation and the promotion of regulatory responses by the microbiota. For instance, monocolonization of germ-free (GF) mice with the human commensal, *Bacteroides fragilis*, induced the development of IL-10-secreting colonic Tregs (Round and Mazmanian, 2010). Moreover, *Clostridium*-colonized GF mice demonstrated a marked increase in the number of CD4^+^ Tregs in the colon (Atarashi et al., 2011). Interestingly, a significant percentage of the Tregs were not positive for Helios, a transcription factor expressed by natural Tregs (Thornton et al., 2010), indicating that these Tregs were locally derived through regulatory signaling cascades (Atarashi et al., 2011). In line with these reports, our work has shown that oral treatment with a novel strain of *Lactobacillus acidophilus* deficient in lipoteichoic acid (LTA) effectively ameliorated inflammation-induced colitis and colonic polyposis, and restored intestinal homeostasis in experimental models (Mohamadzadeh et al., 2011; Khazaie et al., 2012). Nonetheless, despite current advances in the field, the specific signals delivered by microbes to innate immune cells, particularly DCs, to foster tolerance are not completely understood. To this end, this review focuses on the immunomodulating characteristics of specific cell surface components of *L. acidophilus* and discusses potential mechanisms whereby LTA-deficient *L. acidophilus* is able to promote the suppression of pathogenic intestinal autoinflammation.

## *Lactobacillus acidophilus* AND ITS SURFACE LAYER COMPONENTS

Oral consumption of probiotics has been associated with multiple health benefits, including induction of mucus-secreting cells, maintenance of intestinal permeability, production of antimicrobial factors, colonization resistance, and immune cell activation or regulation (Gareau et al., 2010). Attesting to the importance of a well-balanced microflora, several systemic and intestinal disorders are associated with gut dysbiosis or alterations in the intestinal microbial composition (Nishikawa et al., 2009; De Palma et al., 2010; Giongo et al., 2011; Blumberg and Powrie, 2012; Jeffery et al., 2012). Among the beneficial bacteria used to maintain physiological intestinal balance, lactobacilli have been tested in clinical trials with favorable outcomes (Ouwehand et al., 2002). These benefits are, in part, due to induced changes in the immune system, as specific *Lactobacillus* species are known to stimulate DCs to produce stimulatory and regulatory cytokines that direct subsequent T cell responses (Christensen et al., 2002; Mohamadzadeh et al., 2005; Konstantinov et al., 2008). The immunomodulatory effects of lactobacilli are attributed to the interactions between bacterial cell surface components and pattern recognition receptors (PRRs) expressed on innate cells, such as Toll-like receptors (TLRs) and C-type lectins (CLRs; Konstantinov et al., 2008; Mohamadzadeh et al., 2008). Given the species-specific differential signaling of lactobacilli cell surface components, detailed examination of these proteins is imperative for the achievement of tailored immune responses. Dissecting the downstream consequences of host immune cell–microbial interactions is of particular importance in cases where preexisting inflammation or a propensity for inflammatory conditions might be exacerbated or promoted, respectively, by otherwise harmless bacterial constituents.

*Lactobacillus acidophilus*, one of the most widely consumed beneficial microbes (Sanders and Klaenhammer, 2001), is a Gram-positive bacterium that expresses the highly conserved LTA and other surface-exposed (S-layer) molecules, such as the proteins encoded by *slpA*, *slpB*, and *slpX*. S-layers have putative roles in cell adhesion, cell shape determination, as protective barriers, and as anchoring sites for accessory proteins, all of which may contribute to bacterial survival and host–microbial cell interactions within the gastrointestinal tract. Under laboratory growth conditions, the dominant S-layer protein found on *L. acidophilus* is SlpA (Boot et al., 1996), which is coexpressed with the lesser expressed protein SlpX (Goh et al., 2009). On the other hand, SlpB, due to a chromosomal inversion, is only coexpressed with SlpX in a small fraction of laboratory-grown *L. acidophilus* (Boot et al., 1996) or in some mutants devoid of SlpA (Boot et al., 1996; Konstantinov et al., 2008; Goh et al., 2009). While deletion of SlpA leads to decreased binding ability *in vitro* (Buck et al., 2005), the absence of SlpX did not result in morphological changes, reduced adherence to epithelial cells *in vitro*, or increased sensitivity to cellular stresses (Goh et al., 2009). Still, a *L. acidophilus* mutant lacking SlpX and SlpB is cleared faster *in vivo* than the wild-type strain (Zadeh et al., 2012), suggesting that SlpX and SlpB, albeit to a lesser extent, may also contribute to the gastrointestinal interactions of *L. acidophilus*. In terms of immunomodulatory effects, DCs stimulated *in vitro* with a SlpB-dominant strain (SlpA^-^) produced higher levels of the proinflammatory cytokines IL-12 and tumor necrosis factors-alpha (TNF-α) than those challenged with the parental *L. acidophilus* strain (SlpA^+^; Konstantinov et al., 2008), indicating a potential regulatory role for *L. acidophilus* SlpA that could very well account for our recent exciting observations (Mohamadzadeh et al., 2011; Khazaie et al., 2012). Additionally, the SlpA^-^ mutant demonstrated reduced binding to DC-specific ICAM-3-grabbing non-integrin (DC-SIGN), a CLR expressed on DCs, and no differences in the ability to activate TLR2 (Konstantinov et al., 2008), implying that *L. acidophilus* SlpA does not signal to DCs via TLR2. Conversely, *L. helveticus*-derived SlpA, although very similar to *L. acidophilus* SlpA, was recently reported to downregulate inflammation-associated gene expression when tested *in vitro* using an epithelial cell line, but promoted proinflammatory effects in macrophages via TLR2, also *in vitro* (Taverniti et al., 2012). The authors ascribed these discrepancies to differences in the models employed; nonetheless, the *in vivo* role of *L. acidophilus* SlpA remains to be elucidated and is currently under extensive scrutiny in our laboratories to decipher its immunoregulatory effects using a range of experimental animal models.

In contrast, LTA is regarded as the Gram-positive counterpart of the potent and proinflammatory Gram-negative stimulus, lipopolysaccharide (LPS; Sriskandan and Cohen, 1999; Su et al., 2006). LTA is a zwitterionic glycolipid found in the cell wall of many Gram-positive bacterial strains, including *L. acidophilus*, which is believed to facilitate adhesion, colonization, and invasion of host cells (Reichmann and Gründling, 2011). In addition to the likely role of LTA in *Lactobacillus* adhesion to mucosal surfaces, this molecule promotes immune cellular activation via TLR2 signaling, which then activates downstream proinflammatory cytokine signaling cascades (Schwandner et al., 1999; Chiu et al., 2009; Chang et al., 2010; Saber et al., 2011). Notwithstanding, conflicting reports suggested that LTA from certain *Lactobacillus* species induces anti-inflammatory cytokine production (IL-10), and only results in the generation of proinflammatory mediators in preexisting inflammatory conditions [i.e., co-culture with interferon-gamma (IFN-γ); Kaji et al., 2010; Kang et al., 2011]. Taken together, these data contend that the functions of LTA might differ between bacterial species (beneficial lactobacilli versus pathogenic) as well as depend on the status of the local cytokine milieu (steady state versus proinflammatory). However, a caveat of these studies is that the work was performed *in vitro*, which prompts the following question: what is the physiological role of lactobacilli-derived LTA?

## IMMUNE REGULATION INDUCED BY LTA-DEFICIENT *L. acidophilus*

To clarify the *in vivo* effects of *L. acidophilus*-LTA, we recently developed a *L. acidophilus* strain lacking the gene encoding phosphoglycerol transferase, an enzyme required for the biosynthesis of LTA. As opposed to treatment with the wild-type strain, oral inoculation with LTA-deficient *L. acidophilus* not only prevented chemical and pathogenic T cell-induced colitis, but also quickly resolved established colitis, as measured by diminished percent weight loss, lower diarrhea and fecal occult blood scores, and reduced disease activity index (Mohamadzadeh et al., 2011). By the same token, LTA-deficient *L. acidophilus* dramatically reversed colonic preneoplasia in genetically predisposed animals (Khazaie et al., 2012). While protection from colitis in our studies correlated with an increase in IL-10-producing DCs and the number of iTregs (Mohamadzadeh et al., 2011; Khan et al., 2012), polyposis reversal coincided with an overall dampening of local and systemic immunity that was linked with restoration of Treg function and stability (Khazaie et al., 2012). Importantly, proinflammatory Tregs have also been identified in colorectal cancer (CRC) patients (Blatner et al., 2012), further supporting the clinical applicability of LTA-deficient *L. acidophilus* for the treatment of intestinal maladies given its potential ability to prevent the formation of proinflammatory FoxP3^+^RORγt^+^ Tregs.

**FIGURE 1:**
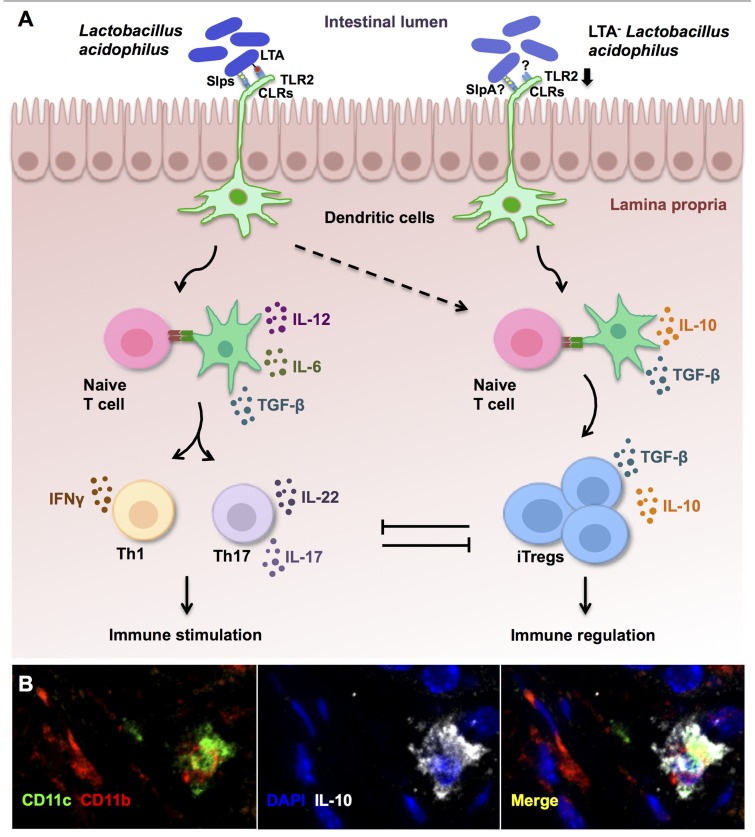
**Immune regulation established by lipoteichoic acid (LTA)-deficient *Lactobacillus acidophilus***. **(A)** In steady state conditions, molecules expressed on the cell surface of *L. acidophilus* activate dendritic cells (DCs) to promote effector Th1 and Th17 responses that are held in check by the accompanying generation of induced regulatory T cells (iTregs). However, in preexisting inflammation or susceptible individuals, immune activation by *L. acidophilus*-LTA exacerbates inflammatory responses and fails to promote immune regulation. Oral intake of mutant strains lacking LTA expression (LTA^-^ *L. acidophilus*) predominantly results in suppression of exacerbated immune responses via the induction of regulatory IL-10-secreting DCs **(B)**, which then promote the conversion of naive T cells into iTregs. **(B)** Confocal microscopy analysis of DCs (CD11c^+^, green; CD11b^+^, red) that produce IL-10 (white) in the colons of healthy control mice after treatment with LTA-deficient *L. acidophilus*.

Moreover, *in vitro* co-culture of DCs with LTA-deficient *L. acidophilus* led to a regulatory DC phenotype, as demonstrated by enhanced IL-10 secretion, low expression of costimulatory molecules, and concomitant decreases in IL-12 and TNF-α production. Alternatively, no beneficial effects could be induced in IL-10^-^^/^^-^ mice *in vivo*, highlighting the important role of this anti-inflammatory cytokine in the control of pathogenic intestinal inflammation in our system, similar to previous findings by others (Asseman et al., 1999; Grangette et al., 2005; Rubtsov et al., 2008). Activation of mitogen-activated protein kinases (MAPK) signaling pathways differentially controls features of both innate and adaptive immune responses (Dong et al., 2002). Favored IL-10 production by regulatory DCs has previously been found to be dependent on extracellular signal-regulated protein kinases 1 and 2 (ERK1/2) activation, while suppressed IL-12 secretion resulted from impaired p38 activation (Qian et al., 2006). Indeed, significant and sustained ERK1/2 activation was measured in the colonic tissues of mice orally treated with LTA-deficient *L. acidophilus*, whereas the wild-type strain promoted p38 phosphorylation (Saber et al., 2011). Furthermore, DC stimulation with LTA-deficient *L. acidophilus* resulted in only weak TLR2-dependent cytokine production and did not enhance the expression of this PRR; these data indicate that LTA is in fact the proinflammatory molecule most strongly associated with TLR2 activation by *L. acidophilus* in DCs, and that the *in vivo* regulatory response noted after LTA-deficient *L. acidophilus* treatment is a direct consequence of its absence. Collectively, the favorable effects of LTA-deficient *L. acidophilus* may be due to weak TLR2 activation and downstream signaling, together with the predominant activation of alternative DC-related PRRs, such as CLRs (Konstantinov et al., 2008), by different surface-associated molecules, including SlpA (summarized in **Figure 1A**).

## CONCLUDING REMARKS AND FUTURE DIRECTIONS

Although the exact signaling pathways whereby LTA-deficient *L. acidophilus* promotes the generation of regulatory DCs and, consequently, iTregs, are currently under intensive investigation, data obtained thus far clearly demonstrate that IL-10-dependent pathways (**Figure 1**) underlie the protective effects of LTA-deficient *L. acidophilus.* In addition, work by others point to SlpA as a potential regulatory molecule in *L. acidophilus* (Konstantinov et al., 2008). Notably, as seen in the wild-type *L. acidophilus* strain, the presence of this S-layer protein alone is not sufficient to counterbalance the proinflammatory actions of LTA. Additional studies performed in our laboratories demonstrated that a mutant strain expressing LTA and SlpA, but not SlpX and SlpB, was unable to afford any protection against colitis (Zadeh et al., 2012). In fact, oral treatment with this LTA^+^SlpA^+^ *L. acidophilus* strain led to a higher number of TNF-α-producing colonic DCs, in addition to sustained IL-12 production by DCs in the colon, when compared to the LTA-sufficient parental strain (Zadeh et al., 2012). These findings may be interpreted to imply that the other S-layer proteins expressed by *L. acidophilus* NCFM also contribute to the regulation of LTA-induced inflammation; however, attempted deletion of SlpA in this strain resulted in slightly lower expression levels of the protein when compared to the parental strain, which then suggests that even small perturbations in the amount of SlpA expressed can exacerbate LTA-mediated inflammation. Consequently, ongoing studies aim to investigate the specific contribution of the S-layer components (i.e., SlpA) to conserve and support gut homeostasis by creating restricted mutant strains of *L. acidophilus* using molecular techniques previously described (Goh et al., 2009) and purifying our protein of interest, SlpA. Thus, the therapeutic value of both SlpA^+^SlpB^-^SlpX^-^LTA^-^ *L. acidophilus* and purified SlpA will be determined *in vivo*.

In other respects, it is likely that LTA-deficient *L. acidophilus* confers additional benefits to the host through mechanisms independent of the immunomodulatory effects mentioned above. For instance, intestinal epithelial cells not only create a protective barrier against invading pathogens, but also sense and interact with microbes through PRRs to influence subsequent innate immune responses (Wells et al., 2011). Accordingly, the status of the mucosal epithelium is central to gastrointestinal health and accumulating evidence indicates that aberrant epigenetic modification of colonic tissue contributes to CRC development (Lao and Grady, 2011). As these changes can arise in the presence or absence of pathogenic intestinal inflammation, we recently tested the effects of LTA-deficient *L. acidophilus* treatment on the epigenetic landscape of the intestinal mucosa and found that this bacterium induced the expression of CRC-associated, epigenetically controlled genes that are often downregulated in cancer-promoting pathogenic conditions (Lightfoot et al., 2012). These important results create a strong position to precisely define the bacterial gene products that may dampen detrimental gut inflammation and protect against inflammatory conditions, including inflammatory bowel disease and colon cancer, not only through immune cell modulation, but also via direct interactions with the gut epithelium.

## Conflict of Interest Statement

The authors declare that the research was conducted in the absence of any commercial or financial relationships that could be construed as a potential conflict of interest.
